# Duck RIG-I CARD Domain Induces the Chicken IFN-*β* by Activating NF-*κ*B

**DOI:** 10.1155/2015/348792

**Published:** 2015-03-30

**Authors:** Yang Chen, Zhengyang Huang, Bin Wang, Qinming Yu, Ran Liu, Qi Xu, Guobin Chang, Jiatong Ding, Guohong Chen

**Affiliations:** ^1^The Key Laboratory of Animal Genetics & Breeding and Molecular Design of Jiangsu Province, Yangzhou University, Yangzhou 225009, China; ^2^College of Animal Science and Technology, Jiangxi Agricultural University, Jiangxi 330045, China

## Abstract

Retinoic acid-inducible gene I- (RIG-I-) like receptors (RLRs) have recently been identified as cytoplasmic sensors for viral RNA. RIG-I, a member of RLRs family, plays an important role in innate immunity. Although previous investigations have proved that RIG-I is absent in chickens, it remains largely unknown whether the chicken can respond to RIG-I ligand. In this study, the eukaryotic expression vectors encoding duRIG-I full length (duck RIG-I, containing all domains), duRIG-I N-terminal (containing the two caspase activation and recruitment domain, CARDs), and duRIG-I C-terminal (containing helicase and regulatory domains) labeled with 6∗His tags were constructed successfully and detected by western blotting. Luciferase reporter assay and enzyme-linked immunosorbent assay (ELISA) detected the duRIG-I significantly activated NF-*κ*B and induced the expression of IFN-*β* when polyinosinic-polycytidylic acid (poly[I:C], synthetic double-stranded RNA) challenges chicken embryonic fibroblasts cells (DF1 cells), while the duRIG-I was inactive in the absence of poly[I:C]. Further analysis revealed that the CARDs (duRIG-I-N) induced IFN-*β* production regardless of the presence of poly[I:C], while the CARD-lacking duRIG-I (duRIG-I-C) was not capable of activating downstream signals. These results indicate that duRIG-I CARD domain plays an important role in the induction of IFN-*β* and provide a basis for further studying the function of RIG-I in avian innate immunity.

## 1. Introduction

The innate immune system is the first line of host defense against viral infection. Host antiviral responses are initiated by the recognition of viral components by host pattern recognition receptors (PRRs), which initiate a signaling cascade that activates IRF3, IRF7, and NF-*κ*B to cooperatively induce transcription of type I IFN genes [1–3]. Type I IFNs further induce downstream proteins, which cause suppression of viral replication, clearance of virus-infected cells, and facilitation of the adaptive immune response [1–3].

Recently, several studies have demonstrated that the mammalian RIG-I-like receptors (RLRs) family presents a strong evidence of acting positive selection [4–7]. RLRs are nucleic acid sensors that activate antiviral innate immune response. These molecules recognize diverse nonself RNA substrates and are antagonized by several viral inhibitors. RIG-I, a member of this family, plays a crucial role in initiating innate antiviral immune responses by sensing intracellular viral RNA, recruiting a specific adaptor protein, IFN-*β* promoter stimulator 1 (IPS-1, also named MAVS, VISA, or Cardif), and activating downstream IFN regulatory factor 3 (IRF3) and IFN regulatory factor 7 [8–11]. Meanwhile, NF-*κ*B signaling induces transcription of type I IFN genes [12]. Most researches on mammals show that RIG-I recognizes a large spectrum of viruses and leads to production of IFN-*β* and expression of downstream IFN stimulated antiviral genes (ISGs) [13].

We previously reported expression of duRIG-I increased in spleen and liver after poly[I:C] challenge [14]. Also, Barber et al. and Huang et al. proposed that duRIG-I deletion might underlie the sensitivity of chicken to avian influenza [15, 16]. Barber et al. have proved that duRIG-I responded to virus by activating IFN-*β* promoter [15]. In this study, we determined functional differences of duRIG-I domains in activating downstream signaling pathways by activation of NF-*κ*B and production of IFN-*β* in chicken cells. The results of the functions of the duRIG-I domains would explore the mechanism of RIG-I and enhance basis researches of avian antiviral immunity.

## 2. Materials and Methods

### 2.1. RNA Extraction and cDNA Synthesis

Total RNA was extracted from each tissue with TRIzol (Invitrogen, USA) according to the manufacturer's instructions, and the quality of the isolated RNA was assessed by visualizing the ribosomal RNA bands after electrophoresis on a 1.0% agarose gel (data not shown). A cDNA synthesis kit (TaKaRa, Japan) was used according to the manufacturer's instructions with 1 *μ*g of total RNA as a template.

### 2.2. Cloning of duRIG-I Gene

According to the reported CDS sequence of the* A. platyrhynchos* RIG-I gene (GenBank accession number EU363349), one pair of primers was designed to amplify the CDS of the RIG-I gene (Table 1). To synthesize duRIG-I cDNA, mRNA isolated from the spleen was used as a template. The cycling parameters were 95°C for 5 min, 35 cycles of 94°C for 45 sec, 68°C for 45 sec, 72°C for 3 min, and a final extension of 72°C for 10 min. The PCR product was cloned into the pMD19-T-simple vector (TaKaRa, Japan) and sequenced.

### 2.3. Construction of Expression Plasmids

Conserved domains within the duRIG-I protein were identified through NCBI (http://www.ncbi.nlm.nih.gov/Structure/cdd/wrpsb.cgi). Based on these results, the primers for the different fragment-containing domains were designed (Table 1). duRIG-I full length (1–933 aa, containing all domains), duRIG-I N-terminal (1–244 aa, containing two CARDs), and duRIG-I C-terminal (192–933 aa, containing helicase and regulatory domains) were inserted into the Acc65I-XbaI sites of pcDNA3.1^+^ with oligonucleotides for a C-terminal 6∗His tag (named duRIG-I-F, duRIG-I-N, and duRIG-I-C, resp.).

### 2.4. Cell Culture and Transfection

UMNSAH/DF-1 cell (Cell Bank of the Chinese Academy of Science, China) was a spontaneously immortalized chicken cell line derived from 10-day-old East Lansing Line (ELL-0) eggs. We cultured the cells as described previously in complete growth medium—Dulbecco's modified eagle medium (GIBCO, USA) and 10% fetal bovine serum (GIBCO, USA)—at 39°C in a humidified 5% CO_2_/95% air incubator. These cells are adherent with a fibroblast-like morphology.

For western blotting analysis and ELISA, DF-1 cells seeded in 24-well plates were grown overnight to 80–90% confluence prior to transfection with 1 *μ*g/well of plasmid (pcDNA3.1^+^, control) using Lipofectamine 2000 (Invitrogen, USA) according to the manufacturer's protocol. After 24 h, cells were stimulated by 10 *μ*g/mL poly[I:C]. Then, the culture supernatant and cells were collected 12 h later.

For the dual-luciferase reporter assay, DF-1 cells seeded in 96-well plates were grown overnight to 80–90% confluence prior to transfection with 0.2 *μ*g recombinant plasmid, 0.05 *μ*g pNF-*κ*B-Luc (no eukaryotic selection, Agilent, USA), and 0.01 *μ*g pRL-TK internal control vector (Promega, UK)/well (pcDNA3.1^+^, control) using Lipofectamine 2000 according to the manufacturer's protocol. After 24 h, cells were stimulated by 10 *μ*g/mL poly[I:C], and luciferase levels were measured 12 h later.

### 2.5. Luciferase Reporter Assay

Luciferase activity was measured with a multifunctional microplate reader (Synergy 2, USA) using a Dual-Luciferase Reporter Assay kit (Promega, UK) and normalized to the activity of an internal control vector, pRL-TK.

### 2.6. ELISA and Western Blotting Analysis

Supernatants were collected after centrifugation at 12000 g for 20 min at 4°C and subjected to a chicken IFN-*β* ELISA (American Research Group Inc., USA). The concentrations of IFN-*β* in the samples were measured with a multifunctional microplate reader (Tecan Infinite M200 PRO, Switzerland) and determined by comparing the O.D. of the samples to the standard curve.

Cells were lysed with RIPA lysis buffer (Solarbio, China) for western blot analysis. The concentration of protein was determined with a BCA protein assay reagent (Thermo Scientific, USA). Protein samples were separated by 10% SDS-PAGE and transferred to a nitrocellulose membrane (BioRad, USA). The membranes were probed with the following antibodies against the His tag (Millipore, USA).

### 2.7. Statistical Analysis

The data were subjected to analysis of variance, and the means were assessed for significant differences using the two-tailed Student's *t*-test. The results are presented as the mean ± SE, at the two significant levels of ^∗^ *P* < 0.05 and ^∗∗^ *P* < 0.01. All statistical analyses were performed in SPSS (version 21).

## 3. Results

### 3.1. Cloning of the Different Conserved Domains of duRIG-I

The full-length CDS sequence of duRIG-I was synthesized from the total RNA of spleen using RT-PCR (GenBank accession number JQ946323) and its homology with known sequence reach 99% (GenBank accession number EU363349). Based on this sequence, the duRIG-I protein was predicted to have conserved domains typical of RLRs through NCBI (http://www.ncbi.nlm.nih.gov/Structure/cdd/wrpsb.cgi), which was consistent with multiple crystal structures of human RIG-I [17, 18]. The conserved domains deduced from the amino acid sequence included two CARDs (residues 2–90 and 99–184), a DEXDc (DEAD/DEAH box helicase domain, residues 261–413), a HELICc (helicase superfamily C-terminal domain, residues 607–742), and an RD (regulatory domain, residues 807–926) (Table 2).

### 3.2. Construction and Identification of duRIG-I Domain Recombinant Plasmids

**Figure 1:**
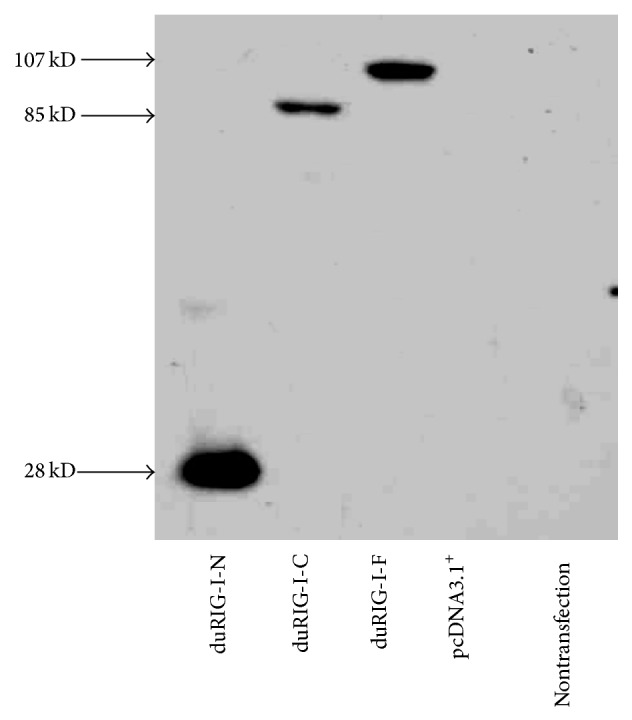
Expression of different mutants of* duRIG-I* in DF1 cells measured by western blot. DF1 cells were transfected with the duRIG-I-F, duRIG-I-N, duRIG-I-C, and pcDNA3.1^+^ plasmids (1 *μ*g each) for 24 h before western blot analysis. Cell lysates were separated by SDS-PAGE, and different mutants of duRIG-I-His were detected with mouse monoclonal antibodies against the His tag. The protein molecular weights (minus the size of the 6∗His tag) are labeled on the left side of the figure. pcDNA3.1^+^–transfected DF-1 was served as a negative control and nontransfection was served as a blank control.

To explore the functions of the different domains, we cloned different duRIG-I mutants and constructed the eukaryotic expression plasmids duRIG-I-F, duRIG-I-N, and duRIG-I-C. Amplification products were analyzed by 1.0% gel electrophoresis and sequenced (data not shown). Then, these recombinant plasmids were transfected into DF1 cells, and western blot detection was carried out 24 h after transfection. DF1 cells were transfected with pcDNA3.1^+^ as a negative control. We found that the His fusion proteins of different domains are expressed in DF1 cells, as shown in Figure 1.

### 3.3. Functional Analysis of the Induction of IFN-*β* by Different Domains of duRIG-I

**Figure 2:**
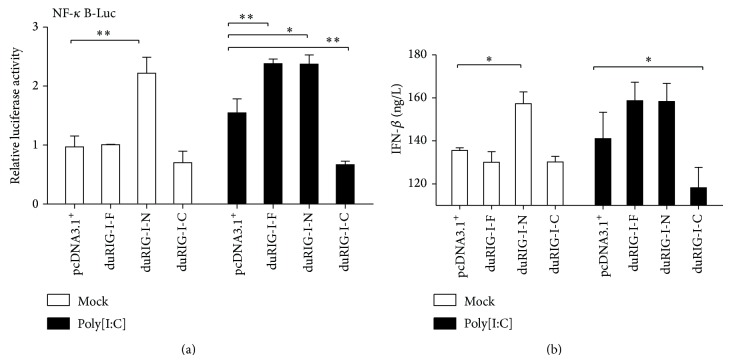
Characterization of the effect of different domains of duRIG-I on the induction of IFN-*β*. (a) DF1 cells were transiently transfected with reporter constructs containing pNF-*κ*B-Luc and internal control vector pRL-TK together with empty vector pcDNA3.1^+^, duRIG-I-F, duRIG-I-N, and duRIG-I-C. The effector/reporter/internal control ratio was 4 : 1 : 1/15. Transfected cells were mock treated (Mock), treated with poly[I:C] for 12 h, were analyzed by the dual-luciferase assay. The data represent relative luciferase activity, normalized to Renilla luciferase activity. The error bars represent SE of triplicate transfections. (b) IFN-*β* in the culture medium was quantified by ELISA after collection of the culture supernatant.

To characterize the domains of duRIG-I and their roles in type I interferon signaling pathway in chicken, the key transcription factor NF-*κ*B was chosen to evaluate induction of IFN-*β*. The results show that duRIG-I was inactive in the absence of poly[I:C] (Figure 2). DuRIG-I was activated by the transcription factor NF-*κ*B significantly and induced the expression of IFN-*β* in poly[I:C]-treated cells. The duRIG-I-N (containing the two CARDs) activated NF-*κ*B and increased IFN-*β* production regardless of poly[I:C] treatment. The CARD-lacking duRIG-I (duRIG-I-C) was not capable of activating downstream signals and inhibited poly[I:C]-induced IFN-*β* production. Thus, the conserved domains play different roles in the activation of NF-*κ*B and induction of IFN-*β* when dsRNA are present in cells.

## 4. Discussion

RIG-I senses viral RNAs and triggers innate antiviral responses through induction of type I IFNs and inflammatory cytokines [19, 20]. RIG-I possesses a precise mechanism of antiviral immune responses. The current study suggests that its domains may interact with each other and that the ligand RNA interaction induces critical conformational changes to trigger biological signals [21]. As we known, human RIG-I contains two repeats of CARD-like motif at its N-terminus [22–24]. The signal leading to the induction of type I interferon upon activation was transferred to downstream pathways via the adaptor molecule IPS-1 through the CARD-CARD interaction [25]. The Lys 63-linked ubiquitination of huRIG-I CARD was crucial for the RIG-I signaling pathway to elicit host antiviral innate immunity [26]. Most functional researches on the mechanism of RIG-I domains focused on mammals, rarely on avian [20, 27–30].

Barber et al. have predicted that RIG-I deletion might underlie the sensitivity of chickens to avian influenza [15]. The cell of chicken has provided a preferred model for the study of the mechanisms of duRIG-I function on avian innate immunity. Barber et al. described the activation of innate immune genes downstream of duRIG-I signaling in chicken cells by microarray and confirmed by qPCR of interferon stimulated genes including* Mx*,* PKR*, and* IFN-β* [31]. This result indicates that duRIG-I plays a key role in the induction of type I interferon, falling in line with historical findings [15]. However, it is not clear how duRIG-I induces avian innate immune response. In this study, Poly[I:C] was used to mimic the induction of the classical antiviral response by potently inducing IFN-*β* production. There are differences between Mock and poly[I:C]-induction for the empty vector pcDNA3.1^+^, but not significant. The differences may be caused by MDA5, which also recognize poly[I:C] and prefer to long one (>1 kb) [32]. The emphasis is on the structural characterization of duRIG-I and its role in type I interferon signaling in a chicken cell line.

N-terminal duRIG-I, in which residues 1–244 encode the tandem CARDs alone, without the ATP binding site, was constructed. Before and after treatment with poly[I:C], the expression of the CARDs promoted IFN-*β* production. On the other hand, for full-length duRIG-I, the activity of NF-*κ*B was increased significantly by comparing the luciferase levels driven after poly[I:C] treatment. Our results show that IFN-*β* secretion increased compared with empty control when stimulated with poly[I:C], but the effect was not significant. There might be a better ligand to activate RIG-I. Kowalinski et al. crystallized duRIG-I and further demonstrated that 5′triphosphate double-stranded RNA (5′ppp-dsRNA) activates RIG-I [17]. The luciferase levels of C-terminal duRIG-I were always low and duRIG-I-C inhibits the poly[I:C]-induced activity. The effects on IFN-*β* induction are consistent with luciferase reporter assays. Previous reports have indicated that the RIG-I molecule is folded in an inactive state with the CARD occluded by the C-terminus in the absence of virus RNA. RIG-I signaling is negatively regulated through internal C-terminal domains. The C-terminus possesses the repressor domain (RD) and interacts with the CARD and a helicase domain. According to published reports, the RD could block RIG-I–mediated signaling [22, 24, 33]. Some historical studies hold that mutation of the ATP binding site (K270A) abolishes the antiviral function of RIG-I; in other words, the ATP binding site is essential [24]. But our findings support the previous research indicating that the tandem CARDs without the ATP binding site also could induce IFN-*β* production [34]. The tandem CARDs of duRIG-I are responsible for activating downstream signaling pathways that mediate poly[I:C]-induced IFN-*β* production. In other words, duRIG-I CARD domain induces the chicken IFN-*β* by activating NF-*κ*B. However, the new study shows that there are not ubiquitinated sites in duck CARD domains for activation of duRIG-I by TRIM25 [35]. Different with those studies on human, further research is needed to understand the mechanism of duRIG-I.

In summary, in the absence of poly[I:C], the sensor molecule is folded in an inactive state. When poly[I:C] infects the cell, the conformation of duRIG-I changes. The CARD interacts with downstream signaling molecules, leading to type I IFN transcription by activating NF-*κ*B. Those results offer some insights into the mechanism of duRIG-I function and provide a basis research of avian innate immunity.

## Figures and Tables

**Table 1:** Primer information for vector construction and RT-qPCR.

Name of primer	Sequences (5′→3′)	Annealing temperature (°C)	Note
RIG-I-Full-F RIG-I-Full-R	CCG*GGTACC*ATGACGGCGGACGAGAAGCGGAG TGC*TCTAGA*CTA*atgatgatgatgatgatg*AAATGGTGGGTACAAGTTGGAC	68	CDS amplification

RIG-I-Full-F RIG-I-N-R	CCG*GGTACC*ATGACGGCGGACGAGAAGCGGAG TGC*TCTAGA* **CTA** *atgatgatgatgatgatg*CTTTGTTTCATAGACAGGTGGAGGTTTGC	68	N-terminal amplification

RIG-I-C-F RIG-I-Full-R	CCG*GGTACC* **ATG**GCCAAAGATGTTGACAGTGAAATGA TGC*TCTAGA*CTA*atgatgatgatgatgatg*AAATGGTGGGTACAAGTTGGAC	68	C-terminal amplification

*Note.* The underlined italics indicate the enzyme cutting site, the lowercase italics indicate the 6∗His tag sequence, and the bold letters indicate additional termination and initiation codons.

**Table 2:** The features of retinoic acid inducible protein I in the duck.

Name	Region/site (aa)	Note
CARD_RIG-I_1	2–90	Caspase activation and recruitment domain found in RIG-I, first repeat
CARD_RIG-I_2	99–184	Caspase activation and recruitment domain found in RIG-I, second repeat
DEXDc	261–413	DEAD-like helicases superfamily. A diverse family of proteins involved in ATP-dependent RNA or DNA unwinding. This domain contains the ATP-binding region
HELICc	607–742	Helicase superfamily c-terminal domain; associated with DEXDc-, DEAD-, and DEAH-box proteins; this domain is found in a wide variety of helicases and helicase related proteins
RIG-I_C-RD	807–926	C-terminal domain of RIG-I
ATP binding site	270–274, 706, 727, 731, 733	Chemical binding
Putative Mg++ binding site	375–378	Ion binding
RNA binding site	511-512, 515, 519	Nucleotide binding
RD interface	519, 522-523, 536-537, 540	Polypeptide binding
Helicase domain interface	554-555, 557–560, 563, 567	Polypeptide binding
Nucleotide binding region	636–639, 663-664, 698–700	Chemical binding

*Note.* Protein_id: “ACA61272.1”; protein full length: 1–933 aa; organism: “*Anas platyrhynchos.*”
